# COVID‐19 and thalassemia beta major in splenectomized patient: Clinical case progression and literature review

**DOI:** 10.1002/ccr3.3345

**Published:** 2020-09-12

**Authors:** Lina Okar, Mohammad Ali, Jabeed Parengal, Mohamed A. Yassin

**Affiliations:** ^1^ Department of Medical Education Hamad Medical Corporation Doha Qatar; ^2^ Department of Medicine Infectious Disease Section Hamad Medical Corporation Doha Qatar; ^3^ Department of Medical Oncology, Hematology Section National Center for Cancer Care & Research Hamad Medical Corporation Doha Qatar

**Keywords:** blood transfusion, COVID‐19, hemoglobinopathies, iron chelation therapy, splenectomy, thalassemia beta major

## Abstract

Although the possibility of asymptomatic course for COVID‐19 infection in splenectomized thalassemia beta major patients is present, screening them for COVID‐19 is important as the progression is still not clear.

## Graphical abstract

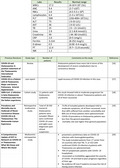

## 1 INTRODUCTION

Thalassemia is one of the most common hemoglobinopathies. The exact course of COVID‐19 infection in those patients is still not clear. We report a case of 25‐year‐old Pakistani woman with beta‐thalassemia major (BTM) on regular blood transfusion, status postsplenectomy who had an asymptomatic course of COVID‐19 infection.

SARS‐CoV‐2 is the novel coronavirus that was designated by the World Health Organization in February 2020 as the reason for COVID‐19 infection that spread rapidly worldwide causing an epidemic (WHO).^1^ Clinical course for COVID‐19 infection might be asymptomatic or vary from typical presentations like fever, malaise, and respiratory symptoms to atypical presentations as gastrointestinal symptoms.^2^

Thalassemia is one of the most common hemoglobinopathies. The main pathophysiology behind thalassemia is the reduction in alpha or beta chain that contributes to hemoglobin formation. Thus, red blood cells will be damaged leading to ineffective erythropoiesis and hemolytic anemia. Thalassemia disorders are classified upon either their genotype of beta‐globin chain or their clinical course as transfusion‐dependent or nondependent. Beta‐thalassemia major is the most severe form of thalassemia, and it is transfusion‐dependent and manifests early in childhood.^3^, ^4^

To the best of our knowledge, the exact course of COVID‐19 infection in thalassemia patients is still not clear. By reviewing the literature, it was mentioned that patients with thalassemia whether they are transfusion‐dependent or independent may be at higher risk of COVID‐19 complications than others because of the increased risk of comorbidities due to iron overload and splenectomy.^5^, ^6^

We report a 25‐year‐old Pakistani woman with a known case of transfusion‐dependent beta‐thalassemia major who tested positive for COVID‐19 infection by screening. The hospital course was smooth though she had a splenectomy and evidence of iron overload with MRI heart and liver.

The progression of COVID‐19 infection in hemoglobinopathy patients in general and thalassemia in specific is not well recognized and still an area of debate and under investigation. Our case highlights the possibility of a good prognosis in thalassemia patients even with the existence of splenectomy.

## 2 CASE PRESENTATION

A 25‐year‐old Pakistani lady with transfusion‐dependent beta‐thalassemia major visited hospital for her scheduled blood transfusion during the pandemic of COVID‐19 in Qatar. She had no fever, respiratory, or gastrointestinal complaints, and no travel history or sick contact with a confirmed COVID‐19 case. Last hospital admission for blood transfusion was 2 months before. Her past medical history was remarkable for open splenectomy and cholecystectomy on 22 January 2019 as a part of the pre–bone marrow transplantation plan. On admission, vitals were temperature 37°C, blood pressure 110/70 mm Hg, respiratory rate 18/min, heart rate 90 per min, and saturation 99% on room air. Physical examination was unremarkable. Due to the current COVID‐19 pandemic and as per our local strategy to eliminate COVID‐19 infection among the population nasopharyngeal swab to screen for COVID‐19, PCR was done and tested positive with CT value (RdRp gene 26.73 and E gene 27.26) which means the patient is still contagious, as per local guidelines (CT value more than 30 is needed to consider not contagious). Thus, indicated investigations in this case were ordered.

**FIGURE 1:**
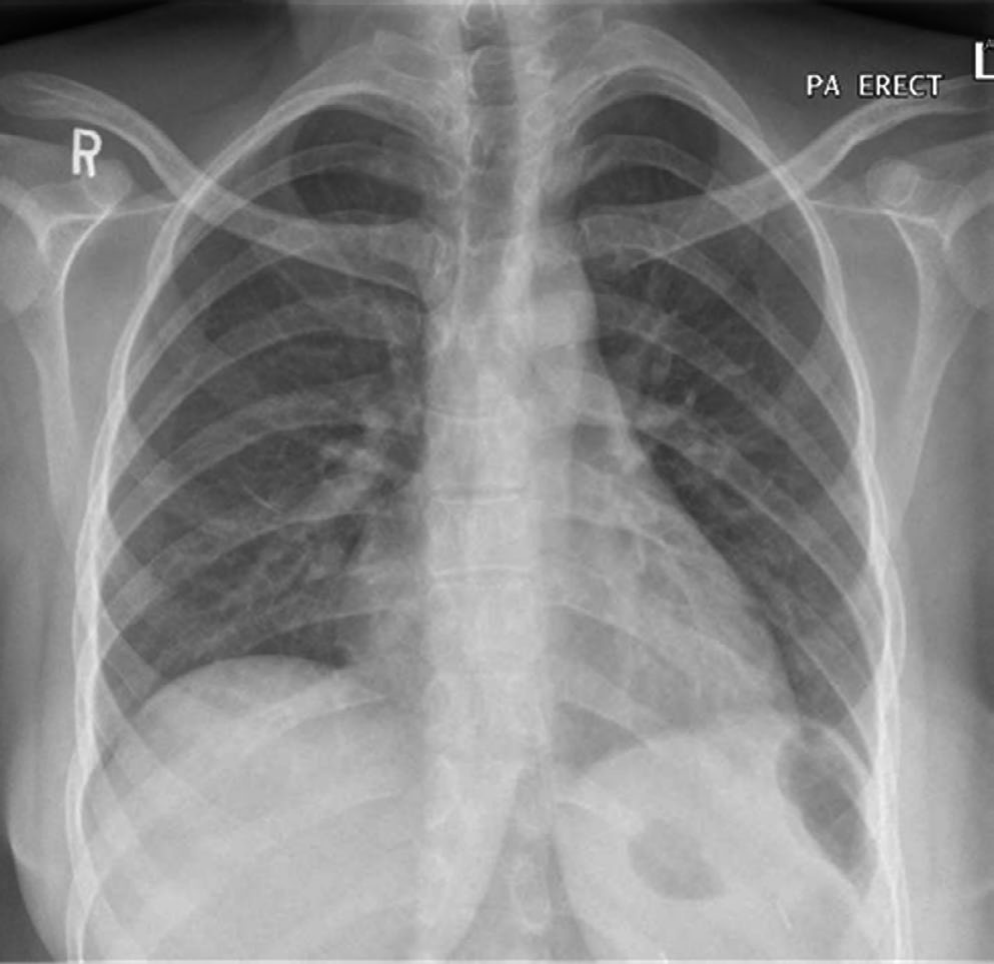
Chest X‐ray

Chest X‐ray (Figure 1) showed no signs of pneumonia, ECG was unremarkable with normal corrected QT and no indicators for any ischemic changes, and basic laboratory tests results were as follows (Table 1): Last hemoglobulin electrophoresis was 3 months before admission. The last magnetic resonance imaging of heart and liver to assess for iron overload was on 2019 and showed only moderate iron overload in the liver.

**Table 1:** XXXX

Laboratories	Results	Normal range
WBCs	17.3	(4‐10 × 10^3^/UL)
ANC	9.4	(2‐7 × 10^3^)
RBCs	3.6	(4.5‐5.5 × 10^6^)
Lymphocytes	6.4	(1‐3 × 10^3^/UL)
PLT	599	(150‐400 × 10^3^/UL)
ALT	73	(0‐33 U/L)
AST	36	(0‐32 U/L)
TB	15	(0‐21 Umol/L)
Urea	2.9	(2.1‐8.8 mmol/L)
Creatinine	49	(44‐80 Umol/L)
CRP	2.0	(0‐5 Umg/L)
Ferritin	3.214	(8‐252 mcg/L)
D‐dimer	0.5	(0.00‐0.4 mcg/L)
PT	12.4	(9.7‐11.8 s)
INR	1.2	

She was admitted for four days and received blood transfusion as planned, and because she was diagnosed previously as COVID‐19, all necessary infection control measures were taken by the medical staff, she received two units of O positive packed red blood cells without any complications, and hemoglobin increased from 8.8 mg/dL to 11.5 gm/dL. Our patient stayed in the ward to monitor her COVID‐19 infection progression and reassess her after blood transfusion. She was stable for the whole hospital course, and she did not develop any respiratory symptoms or need any oxygen supplementation. No specific treatment for COVID‐19 infection was offered, no anticoagulation prophylaxis was given as the patient was ambulating, and decision was made after balancing the risks and benefits. After that, she transferred to a quarantine facility. Upon discharge, the plan was to take deferasirox at dose 1080 mg daily and schedule blood transfusion appointment after 1 month. After 30 days, repeated COVID‐19 PCR was negative.

## 3 DISCUSSION

Coronaviruses are a group of viruses that belong to the Orthocoronavirinae family. The recent viral disease outbreaks that manifested as severe acute respiratory syndrome (SARS) are caused by SARS‐associated coronavirus (SARS‐CoV), Middle East respiratory syndrome (MERS) caused by the MERS coronavirus (MERS‐CoV), and most recently, the coronavirus disease 19 (COVID‐19) caused by SARS coronavirus 2 (SARS‐CoV‐2). SARS‐CoV‐2 was first isolated in Wuhan, China; then, it spread rapidly causing a worldwide pandemic.^7^

Thalassemia's beta is a hereditary disorder due to a defect in hemoglobin synthesis, thus results in persistent hemolysis and increases the premature death of RBCs in bone marrow.^8^ Chronic and severe anemia, as well as bone marrow expansion, happens because of this condition.^9^

If left untreated, thalassemia can lead to major complications. Thalassemia disorders are classified upon clinical and genetic background to beta‐thalassemia major (BTM), beta‐thalassemia intermediate (BTI), or beta‐thalassemia minor (carriers). In practice, β‐thalassemia has been categorized into two main categories either transfusion‐dependent β‐thalassemia (TDT) or non–transfusion‐dependent β‐thalassemia (NTDT).

Unfortunately, once patients are diagnosed with BTM they need a lifelong blood transfusion and thereby suffering from its related complication.^9^, ^10^, ^11^, ^12^

Even though blood transfusion is considered the cornerstone in the management plan for BTM, it is still a problematic issue because of the major complications like iron overload, transfusion‐transmitted infections (TTIs), and antibody formation. Thus, iron chelation therapy (ICT) is of an important value in increasing life span and reducing morbidities related to iron overload.^13^ Our patient was on scheduled blood transfusion along with iron chelation therapy, and COVID‐19 infection was asymptomatic. Whether this smooth clinical course could be attributed to this therapy is an issue lacks current topic, yet it is worthy to highlight the importance of providing BTM patients with ICT whenever is indicated.

Several factors increase the risk of infections in thalassemia disorders especially immune system dysfunction, iron overload, and multiple blood transfusions. In addition, splenectomy and adrenal hypofunction might play a role in increased infections especially in older patients.^14^

Another point to consider is that patients with regular blood transfusion thought to be high risk for COVID‐19 infection due to their recurrent visits to the healthcare facilities.^15^ Our patient was on regular blood transfusions. Thus, we emphasize the importance of screening for COVID‐19 in thalassemia patients during this pandemic even if they are asymptomatic.

The exact clinical course of COVID‐19 infection in BTM patients is not well recognized yet. Conflicting data are available (Table 2).^16^, ^17^, ^18^, ^19^, ^20^

**Table 2:** Literature review summary

Previous literature	Study type	Number of patients	Comments on the study	Section references
COVID‐19 and thalassaemia: A position statement of the Thalassaemia International Federation	Review		Thalassemia patients have more risk in terms of the development of severe complications due to coronavirus disease.	16
COVID‐19 in a patient with β‐thalassemia major and severe pulmonary arterial hypertension	Case report		Rapid recovery of COVID‐19 infection in this case.	17
SARS‐CoV‐2 infection in beta thalassemia: Preliminary data from the Italian experience	Cohort study	11 patients with thalassemia syndrome	The result showed mild to moderate progression for COVID‐19 infection in eleven thalassemia patients and all of them recovered.	18
Prevalence and mortality due to outbreak of novel coronavirus disease (COVID‐19) in β‐Thalassemias: The Nationwide Iranian Experience	Retrospective multicentric study.	A total of 18 350 Iranian β‐thalassemia patients were included: fifteen were confirmed COVID‐19 cases and eight cases diagnosed upon clinical suspension.	73.9% of included patients developed mild to moderate symptoms, all of them recovered, more than 60% suffered from another comorbidity and 80% out of those patients were splenectomized. COVID‐19 prevalence in thalassemia patients was less than the general population. Mortality rate was higher than general population.	19
A comprehensive update of ICET‐A network on COVID‐19 in Thalassemias: What we know and where we stand	Multicentric retrospective study		Presented a preliminary data on COVID‐19 infection with hemoglobinopathies. The study included patients from 17 centers from 10 countries who had TM, TI, or SCD with confirmed COVID‐19 infection, 4 patients with thalassemia are splenectomized. 70% of symptomatic patients with COVID‐19 were hospitalized. Associated comorbidities aggravated the severity of COVID‐19 and lead to poor prognosis regardless of their age. No evidence till now if asplenia increase the risk of having severe infection.	20

COVID‐19 has been associated with thrombosis and increased need for anticoagulation. However, patients with hemoglobinopathies are at higher risk for bleeding; thus, they should be monitored carefully for bleeding. To decide whether anticoagulation is indicated or not, physicians should balance the risks and benefits for every patient. In our patient, as she had asymptomatic COVID‐19 infection course, no risk for thrombosis as per history, examination, and primary investigations, no anticoagulation was given.^21^

In our case, we presented asymptomatic COVID‐19 infection diagnosed by screening in a splenectomized BTM patient who was on a scheduled blood transfusion plan and ICT during the epidemic in Qatar.

## 4 CONCLUSION

Beta‐thalassemia major is one of the most common hemoglobinopathies. Patients with this disorder exhibit a wide range of presentation and complications mostly from the iron overload. ICT is an important way to reduce complications. One of the worthiest questions to ask nowadays is whether those patients should be considered high risk for COVID 19 infection and its complications during this pandemic or not. A final decision is yet to be made. We believe it is important to screen for COVID‐19 infection in thalassemia patients as they might be asymptomatic.

We reported a smooth clinical course for COVID‐19 infection in a splenectomized female with BTM.

Further data, of course, need to be collected to know the exact course of COVID‐19 in Thalassemia patients, also if compliance with iron chelation therapy has any role to play in minimizing the presenting symptoms.

## CONFLICT OF INTEREST

None declared.

## AUTHOR CONTRIBUTIONS

LO, MA, and MY: were involved in data collection, analysis, and interpretation. LO: wrote the manuscript. MA, MY, and JP: critically revised the manuscript.

## ETHICAL APPROVAL

Consent was obtained from the patient. Case approved by HMC Medical Research Center.
